# Application of penalized linear regression methods to the selection of environmental enteropathy biomarkers

**DOI:** 10.1186/s40364-017-0089-4

**Published:** 2017-03-09

**Authors:** Miao Lu, Jianhui Zhou, Caitlin Naylor, Beth D. Kirkpatrick, Rashidul Haque, William A. Petri, Jennie Z. Ma

**Affiliations:** 1 Department of Statistics, University of Virginia, Charlottesville, USA; 2 Division of Infectious Diseases, School of Medicine, University of Virginia, Charlottesville, USA; 3 Department of Medicine and Vaccine Testing Center, University of Vermont College of Medicine, Burlington, USA; 4 The International Centre for Diarrhoeal Disease Research, Bangladesh (icddr,b), Dhaka, Bangladesh; 5 Division of Biostatistics, Department of Public Health Sciences, University of Virginia, Charlottesville, USA

**Keywords:** Biomarker selection, Penalized linear regression, Correlated covariates, Malnutrition, Environmental enteropathy

## Abstract

**Background:**

Environmental Enteropathy (EE) is a subclinical condition caused by constant fecal-oral contamination and resulting in blunting of intestinal villi and intestinal inflammation. Of primary interest in the clinical research is to evaluate the association between non-invasive EE biomarkers and malnutrition in a cohort of Bangladeshi children. The challenges are that the number of biomarkers/covariates is relatively large, and some of them are highly correlated.

**Methods:**

Many variable selection methods are available in the literature, but which are most appropriate for EE biomarker selection remains unclear. In this study, different variable selection approaches were applied and the performance of these methods was assessed numerically through simulation studies, assuming the correlations among covariates were similar to those in the Bangladesh cohort. The suggested methods from simulations were applied to the Bangladesh cohort to select the most relevant biomarkers for the growth response, and bootstrapping methods were used to evaluate the consistency of selection results.

**Results:**

Through simulation studies, SCAD (Smoothly Clipped Absolute Deviation), Adaptive LASSO (Least Absolute Shrinkage and Selection Operator) and MCP (Minimax Concave Penalty) are the suggested variable selection methods, compared to traditional stepwise regression method. In the Bangladesh data, predictors such as mother weight, height-for-age z-score (HAZ) at week 18, and inflammation markers (Myeloperoxidase (MPO) at week 12 and soluable CD14 at week 18) are informative biomarkers associated with children’s growth.

**Conclusions:**

Penalized linear regression methods are plausible alternatives to traditional variable selection methods, and the suggested methods are applicable to other biomedical studies. The selected early-stage biomarkers offer a potential explanation for the burden of malnutrition problems in low-income countries, allow early identification of infants at risk, and suggest pathways for intervention.

**Trial registration:**

This study was retrospectively registered with ClinicalTrials.gov, number NCT01375647, on June 3, 2011.

## Background

High dimensional data analysis has become common and important in biomedical studies. For example, tens of thousands of molecular expressions are potential predictors in microarray data; hundreds of thousands of single nucleotide polymorphisms (SNPs) are possibly associated with the clinical outcome of interest in genome-wide association study [1]. To deal with large number of covariates or predictors, one common approach is testing the association between each covariate and the outcome of interest through univariate regression model; a subset of those covariates are then selected based on their significance for subsequent multivariable analysis. This framework is a common method in biomedicine for variable selection, but it can be a great challenge when the number of covariates is large in massive datasets. Also, prediction accuracy and interpretability are two main drawbacks for this traditional regression analysis [2]. Another widely used method in variable selection is regression with stepwise selection, where the choice of predictive variables is carried out by an automatic procedure. However, the essential problems with such method remain, that is, the parameter estimates tend to be highly biased in absolute values, their standard errors tend to be incorrect, and *p*-values tend to be too low due to multiple comparisons and are difficult to correct [3].

Penalized regression methods such as LASSO (Least Absolute Shrinkage and Selection Operator [4]) and SCAD (Smoothly Clipped Absolute Deviation [5]) have been developed to overcome the limitation of traditional variable selection methods when the number of covariates is large. However, these penalized regression methods remain less familiar to biomedical researchers, but start to gain more attentions in clinical applications [6–10]. In this study, we reviewed several penalized linear regression methods along with the regression method with stepwise selection, presented some common tuning parameter selection criteria, and compared their numerical performance through a simulation study which has similar setting as our motivating clinical example.

Our study was motivated by the data from a birth cohort study, the PROVIDE (Performance of Rotavirus and Oral Polio Vaccines in Developing Countries) study. The PROVIDE study was aimed at investigating oral vaccine efficacy and the impact of environmental enteropathy (EE) on vaccine failure and malnutrition in Bangladesh children. EE, also known as tropical enteropathy or environmental enteric dysfunction, is a subclinical condition or gut disorder caused by constant fecal-oral contamination and resulting in blunting of intestinal villi and intestinal inflammation [11]. EE is prevalent among inhabitants of low-income countries living in environments with poor sanitation and hygiene, where diarrhea and respiratory infections are the leading causes of death in children under age 5. However, both non-invasive tests and effective interventions for EE are lacking [12]. In the PROVIDE study, a large and comprehensive set of non-invasive biomarkers were developed from fecal and blood samples that were collected surrounding the time of vaccination, and some of them were highly correlated. One of the primary study interests was to identify non-invasive EE biomarkers associated with malnutrition and vaccine responses. By investigating the association of these earlier risk factors and biomarkers with child growth, effective intervention strategies for malnutrition can be developed. There are two main challenges in the PROVIDE study: the relatively large number of biomarkers or covariates and the strong correlation among these biomarkers. To overcome these challenges in practice, we prefer to use the penalized linear regression models for the biomarker selection. However, given many variable selection methods available in the literature, which are most appropriate for EE biomarker selection remains unclear. Our objective was to assess the performance of different penalized linear regression methods numerically through a simulation study under different variable selection scenarios. The suggested methods from simulations were applied to the PROVIDE study cohort.

## Methods

In this section, we first describe the PROVIDE study and the data collection. Then, we introduce the penalized linear regression models along with their pros and cons. Next we specify the simulation setting to compare penalized models. Finally, we describe the methods for biomarker data analysis. The simulation results and the biomarker data analysis results are presented in the Results section.

### Data description

The study design, recruitment and follow-up of the PROVIDE cohort were described previously [13]. Briefly, a birth cohort of 700 infants from the Mirpur urban slum in Dhaka, Bangladesh were enrolled and followed for 2 years. The PROVIDE study was a randomized controlled clinical trial with a 2-by-2 factorial design to investigate the efficacy of Rotavirus and Oral Polio Vaccines. During the first 2 years of life, children were monitored through twice weekly household visits by field research assistants and regularly scheduled clinical visits. There was rolling admission of subjects over the first 18 months and the study spanned from May 2011 to November 2014. A comprehensive set of biomarkers were developed from fecal, urine and blood samples that were collected at week 6, 12, 18 and 24 of age. The study was approved by the Ethical Review Board of the ICDDR,B (FWA 00001468) and the Institutional Review Boards of the University of Virginia (FWA 00006183).

The height-for-age z-score (HAZ) at one year old was the outcome of interest in this study. HAZ is an age- and gender-normalized measure of child height using the world health organization (WHO) Multicenter Growth Reference Study Child Growth Standards. HAZ has been considered as the most important measurement for malnutrition in the literature as it captures the long-term cumulative effects of health throughout the childhood and is known to be correlated with later life outcomes [14, 15]. Our interest was to evaluate and identify the significant effects of these earlier risk factors and biomarkers on the child growth. We hypothesized that early infant intestinal and systemic biomarkers, as well as socioeconomic status (SES), nutritional measures, and maternal factors, were significantly associated with HAZ at one year old. All together, a total of 33 biomarkers and clinical risk factors were available, and their descriptive statistics (means and standard deviations) were summarized in Table 1. Note that those listed under the enteric inflammation category were considered to be the EE biomarkers, which were of particular interest. A key challenge in the clinical study was how to identify the informative biomarkers associated with HAZ. Nevertheless, some of these biomarkers were highly correlated, presenting an extra challenge to the data analysis.

**Table 1:** Biomarker list and descriptive summary in PROVIDE study (*N*=512)

Variable category	Biomarker	Child age (week)	Mean (SD)
Enteric inflammation	Myeloperoxidase (MPO)	12	10952.92 (11489.08)
	Calprotectin	12	781.68 (725.30)
	Neopterin	12	2601.90 (2041.17)
	Alpha-1 anti-trypsin (ALA)	12	0.85 (0.71)
	Mannitol in urine	12	0.02 (0.02)
		24	0.02 (0.02)
	Reg1B	6	56.13 (91.12)
		12	80.87 (117.88)
	Days of diarrhea	18	6.22 (10.75)
Systemic inflammation	Ferritin	6	229.42 (153.31)
		18	45.45 (56.39)
	C Reactive Protein (CRP)	6	1.11 (3.83)
		18	2.89 (7.53)
	Soluble CD14	6	1686.90 (630.27)
		18	1967.24 (697.34)
	Endocab lipopolysaccharide (LPS)	6	29.21 (42.25)
		18	11.27 (39.98)
	Log Scale of Activin	6	6.41(1.12)
Nutritional measures	Vitamin D	6	35.58 (18.20)
		18	61.38 (24.17)
	Zinc	6	725.64 (107.58)
		18	771.86 (146.50)
	Retinol binding protein (RBP)	6	24317.52 (11461.94)
		18	29780.83 (15167.70)
	Height for age z score (HAZ)	Birth	-0.90 (0.89)
		18	-1.02 (0.93)
	Weight for age z score (WAZ)	18	-0.82 (1.06)
	Weight for height z score (WHZ)	18	-0.10 (1.01)
	Days of exclusive breast milk feeding	18	95.99 (41.71)
Maternal health, SES	Monthly household expenditure	NA	11736.56 (7555.13)
	Monthly household income	NA	13021.23 (9708.96)
	Mother height (cm)	NA	150.38 (5.61)
	Mother weight (kg)	NA	49.36 (9.33)

### Penalized linear regression

**Fig. 1:**
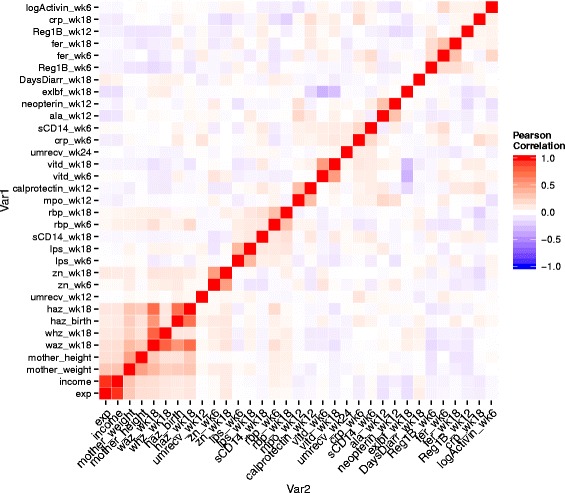
Heatmap of correlation for all biomarkers

To select informative biomarkers and risk factors from 33 available predictors that are associated with HAZ at one year old, traditional methods (univariate regression or stepwise regression) have drawbacks as described in the Background. In practice, since only a small number of factors are truly informative with respect to the response of interest, univariate or multivariable regression analyses could produce biased or false-positive results. In addition, identifying important biomarkers in this case may particularly be challenging because some factors were strongly correlated (Fig. 1). In the PROVIDE data, eight predictors were highly correlated, including monthly household expenditure (exp), income, mother weight, mother height, weight-for-age z-score (WAZ) at week 18, weight-for-height z-score (WHZ) at week 18, HAZ at birth and week 18. In Fig. 1, the heat map of correlation matrix (ordered by correlation coefficients) is plotted. It would be reasonable to assume the correlation structure among these 8 predictors to be autoregressive with order 1 (i.e., AR(1)) and the other 25 covariates to be independent. We were interested in identifying appropriate variable selection methods that can perform well when some of the covariates are highly correlated as in the motivating example.

Consider the linear regression model

1 $$ Y=X\beta+\epsilon,  $$

where *Y* is an *n*×1 vector and *X* is an *n*×*p* matrix. Linear regression is a widely used method to study association between continuous outcome and covariates. In the presence of multiple covariates or predictors, univariate linear regression followed by multivariable linear regression with thresholding *p*-values is the conventional approach in biomedical research. However, given the drawbacks in the traditional approach as discussed in the Background, penalized regression methods have become popular and better alternatives to select variables and estimate regression parameters simultaneously. The regression parameters are assumed to be sparse with some components being zero, while the nonzero components are for the informative variables. Penalized methods shrink the estimates of regression coefficients towards zero relative to the least squares estimates.

A form of the penalized least squares estimator is the minimizer of the following objective function,

2 $$ \frac{1}{2n} \|Y-X\beta\|^{2}+\sum\limits_{j=1}^{p}p_{j} (|\beta_{j}|),  $$

where *β*=(*β*_1_,...,*β*_*p*_) and *p*_*j*_(|·|), is the penalty function, which takes different forms for different methods. The first part of the objective function is the sum of least squares errors, measuring the model goodness of fit. The second part is penalty term, representing model complexity. Specifically, different penalized linear regression models with their pros/cons are summarized in the following.

- LASSO: The *L*_1_ penalty, i.e., *p*_*j*_(|*θ*|)=*λ*|*θ*|, corresponds to the LASSO estimator [4]. Applying the *L*_1_ penalty tends to result in many regression coefficients shrunk exactly to zero and a few other regression coefficients with comparatively little shrinkage. If the pairwise correlation among variables are very high, LASSO tends to select only one variable from the group.
- Elastic Net: The *L*_2_ penalty *p*_*j*_(|*θ*|)=*λ*|*θ*|^2^ leads to a ridge regression, which tends to result in all small but nonzero regression coefficients. A linear combination of *L*_1_ and *L*_2_ penalties is Elastic Net. It enjoys a similar sparsity of representation as LASSO, while encouraging a grouping effect [16].
- SCAD: The continuous differentiable penalty function defined by $p_{\lambda }'(\theta)=\lambda \left \{I(\theta \leq \lambda)+\frac {(a\lambda -\theta)_{+}}{(a-1)\lambda }I(\theta >\lambda)\right \}$, for some *a*>2 and *θ*>0, is the SCAD penalty [5], where *p*_*λ*_(0)=0 and *a*≈3.7 as suggested by Bayesian risk analysis.
- MCP: The Minimax Concave Penalty [17] is defined as $p_{\lambda }'(\theta)=\frac {(a\lambda -\theta)_{+}}{a}$, which translates the flat part of the derivative of SCAD to the origin.
- Adaptive LASSO: a weighting scheme $w_{j}=|\tilde {\beta }_{j}|^{-\gamma }$ for the penalty function of LASSO leads to the Adaptive LASSO with penalty $\sum _{j=1}^{p}w_{j}p_{j} (|\beta _{j}|)$ [18].

Though the penalties above quantify the model complexity differently, they all aim to strike a balance between model goodness of fit and model simplicity, and promote sparse estimates of the regression parameters, where the sparsity indicates variable selection. It is shown in the literature that, SCAD, MCP and Adaptive LASSO enjoy the so-called Oracle properties, compared with LASSO and Elastic Net [1]. That is, SCAD, MCP and Adaptive LASSO select the true model consistently and estimate the nonzero parameters with the asymptotic distribution as if the true model were given.

An important issue in variable selection is to determine the optimal value for tuning parameter *λ*, as its value regulates how many variables are to be selected in practice. When *λ*=0, all variables are selected. When *λ*=*∞*, and if the penalty function satisfies ${\lim }_{\lambda \rightarrow \infty }p_{\lambda } (|\theta |)=\infty $ for *θ*≠0, none of the variables is selected. There are various criteria to select tuning parameter, including Mallow *C*_*p*_ [19], Akaike Information Criterion (AIC) [20], Bayesian Information Criterion (BIC) [21], Generalized Cross-Validation (GCV) [22] and Cross Validation (CV) [2]. Wang et al. showed that the penalized estimator with BIC criterion for selecting tuning parameter achieves the model selection consistency and the Oracle properties [23]. Based on their suggestion, we chose the BIC criterion for all the methods in numerical studies. For Elastic Net, we used grid search for both the proportion (*α*) of *L*_1_ and *L*_2_ penalty and tuning parameter.

### Simulation setup

Simulation studies were designed to assess the relative performance of the different penalized linear regression methods in variable selection with respect to correlation structure and signal-to-noise ratio (SNR). SNR is defined as the ratio of signal power to the noise power, calculated as variance of linear combination of predictors (signal) divided by variance of error (noise). The datasets in the simulation were generated with 500 observations and 33 correlated predictors, which are similar to the PROVIDE cohort data. Also, the coefficients in the simulated models were specified similarly as the estimates from the real data. The traditional linear regression was applied with *p*-value <0.05 as the threshold for the univariate selection of biomarkers. Seven covariates (HAZ at week 18, days of exclusive breast milk feeding until week 18, ferritin at week 6, mannitol in urine at week 12, Myeloperoxidase (MPO) at week 12, mother weight and soluble CD14 at week 18) were informative and selected for the outcome. In simulation, 7 out of 33 covariates were assumed to be nonzero. Based on the real data, the correlation structure within the first eight covariates was assumed as AR(1) with *ρ*=0.5, and as independent for the remaining 25 covariates. We generated 100 simulation datasets from a multivariate normal distribution with mean zero and marginal variance one in the covariance matrix as specified above. To explore the influence of weak/strong correlation and SNR, we further varied *ρ*=0.2,0.5,0.8 and SNR=1, 3, 5.

The key criterion for comparing the performance of different methods is the median relative model error (MRME), as suggested in [5]. For an estimator $\hat {\theta }$ of *θ*, the model error is defined by $ME(\hat {\theta })=(\hat {\theta }-\theta)'\Sigma (\hat {\theta }-\theta)$, where *Σ* denotes the covariance matrix of the corresponding regressors. Then the relative model error (RME) is calculated by $ME(\hat {\theta })/ME(\hat {\theta }_{LS})$, with $\hat {\theta }_{LS}$ denotes the least squares estimator of the overall model. The Oracle estimator is the least squares estimator of the true model. Thus, the RME for the Oracle estimator is calculated by $ME(\hat {\theta }_{LST})/ME(\hat {\theta }_{LS})$, with $\hat {\theta }_{LST}$ denotes the least squares estimator of the true model. The MRME is the median of relative model errors.

True positive (TP) and false positive (FP) provide different perspectives of the performance measure for model comparison. For a given simulated dataset, a variable is selected if its estimated coefficient is nonzero. TP is the average number of nonzero covariates being correctly selected, and FP is the average number of zero covariates being incorrectly selected.

### Biomarker data analysis

Environmental enteropathy is a subclinical enteric condition found in low-income countries that is characterized by intestinal inflammation, reduced intestinal absorption, and gut barrier dysfunction. Among all the biomarkers and risk factors in Table 1, we aimed to identify the important subset associated with HAZ at one year in the PROVIDE study birth cohort.

Given a few dozen of available biomarkers as the potential predictors, traditional variable selection methods such as stepwise regression were not practical or less efficient. Modern penalized linear regression methods reviewed earlier can effectively identify the subset of biomarkers that are truly informative to the response of interest. Based on the relative performance of these penalized regression methods from numerical simulations and the degree of correlations among the biomarkers, the suggested methods were applied to the PROVIDE biomarker data to identify the important biomarkers. We also evaluated the validity of methods through bootstrap sampling, and estimated the percentage of times being selected for each biomarker. Similar subsampling/bootstrapping idea can also be found in [24, 25]. For comparison purpose, the traditional stepwise regression method was also applied to the biomarker data. All the data analyses and simulations were implemented and performed using R 3.2 (http://www.r-project.org/).

## Results

### Simulation results

Table 2 summarizes the MRME, TP and FP by different penalized methods over 100 simulated datasets. When the SNR was low (SNR=1), the SCAD penalty performed the best for all three correlation levels (*ρ*) in the simulation setting. The results of SCAD had relatively larger true negative rates, along with relatively smaller FP, resulting in the smallest MRME (43.51% for *ρ*=0.2, 43.00% for *ρ*=0.5 and 42.13% for *ρ*=0.8). The MRMEs of SCAD were closest to the Oracle estimator compared to other methods. Comparatively, Adaptive LASSO and MCP performed relatively well, having the second and third smallest MRME. Though Elastic Net and Stepwise had relatively large TP, they also had largest FP. When SNR was increased to 3, however, Adaptive LASSO performed the best for all the three *ρ* values with the smallest MRME (54.50% for *ρ*=0.2, 56.64% for *ρ*=0.5 and 56.21% for *ρ*=0.8). MCP and SCAD followed as the second and third, with reasonable large TP and small FP. Elastic Net and Stepwise methods again yielded large FP. Finally, when SNR was increased to 5, Elastic Net and LASSO performed less satisfactory. When the correlation was moderate (*ρ*=0.2,0.5), Stepwise did slightly better than Adaptive LASSO and MCP, but when correlation was large (*ρ*=0.8), Adaptive LASSO performed better than Stepwise and MCP. In most of the cases, stepwise had the smallest FP, but its TP was also relatively low. In the case of correlated variables, stepwise tended to select less for both zero and nonzero variables.

**Table 2:** Comparison of methods with different correlation levels (*ρ*) and signal noise ratio (SNR) from 100 simulation datasets

*ρ*	SNR	Criteria	Stepwise	Elastic Net	LASSO	MCP	SCAD	Adaptive LASSO	Oracle
	1	TP	2.30	4.44	4.48	3.78	4.26	3.74	7.00
		FP	0.30	4.06	4.20	2.30	3.39	2.04	0.00
		MRME (%)	60.40	46.34	46.56	45.04	43.51	45.23	17.93
0.2	3	TP	4.97	6.51	6.55	6.05	6.35	6.08	7.00
		FP	0.35	4.75	4.80	2.04	3.34	2.08	0.00
		MRME (%)	65.26	61.87	62.17	56.00	59.48	54.50	17.93
	5	TP	6.16	6.89	6.88	6.68	6.76	6.71	7.00
		FP	0.41	5.30	5.32	1.93	3.21	2.06	0.00
		MRME (%)	46.40	61.05	61.65	49.76	52.87	47.44	17.93
0.5	1	TP	2.29	4.38	4.41	2.66	4.15	3.67	7.00
		FP	0.34	4.16	4.11	2.38	3.36	2.17	0.00
		MRME (%)	57.76	46.01	46.56	44.03	43.00	44.66	17.89
	3	TP	4.95	6.54	6.54	6.01	6.31	6.00	7.00
		FP	0.39	4.79	4.73	1.99	3.20	2.16	0.00
		MRME (%)	65.52	60.72	62.90	56.98	60.66	56.64	17.89
	5	TP	6.15	6.88	6.87	6.70	6.76	6.72	7.00
		FP	0.38	5.28	5.27	1.82	3.04	2.14	0.00
		MRME (%)	46.56	60.39	61.09	49.18	52.93	48.03	17.89
0.8	1	TP	2.07	4.12	4.05	3.30	3.76	3.34	7.00
		FP	0.58	4.47	4.20	2.25	3.24	2.21	0.00
		MRME (%)	58.85	44.01	45.63	44.16	42.13	42.63	18.04
	3	TP	4.54	6.36	6.32	5.76	6.07	5.78	7.00
		FP	0.52	5.16	4.88	2.03	2.90	2.37	0.00
		MRME (%)	65.93	60.92	63.41	58.36	59.99	56.21	18.04
	5	TP	5.90	6.85	6.83	6.51	6.61	6.59	7.00
		FP	0.49	5.59	5.37	1.73	2.83	2.27	0.00
		MRME (%)	52.57	59.45	62.29	53.52	56.85	50.16	18.04

Oracle estimator is the least squares estimator of the true model, which contains seven nonzero covariates. TP (True Positive) is the average number of nonzero covariates being correctly selected. FP (False Positive) is the average number of zero covariates being incorrectly selected. Median relative model error (MRME) is used to measure the overall performance of different models

In summary, the numerical performance of different variable selection methods in this simulation setting depended on both the strength of correlation among covariates and SNR. When SNR was low, SCAD, Adaptive LASSO and MCP were preferred; when SNR was large, Adaptive LASSO, stepwise and MCP performed similarly, regardless of the low/high correlation. The correlation structure in our simulations is quite common in practice, where some variables are independent while other variables are correlated.

### Environmental enteropathy biomarker analysis

We applied both traditional and suggested penalized regression methods from simulations to the biomarker data in the PROVIDE study. A total of 512 children with all 33 biomarkers available were included in this biomarker data analysis. HAZ at one year of age was the outcome of interest, with mean ± SD as −1.47±1.02. The SNR was estimated at 2.6 in the linear model from our data, and the correlation structure among the first eight covariates as AR(1) with *ρ*=0.5. As suggested by the simulation, Adaptive LASSO, MCP and SCAD would be preferred, while Elastic Net and Stepwise methods performed less satisfactorily. The selection result from real data (Table 3) confirmed this numerical observation.

**Table 3:** Selection results by different variable selection methods

Biomarkers	Variable selection methods					
	Stepwise	Elastic Net	LASSO	MCP	SCAD	Adaptive LASSO
HAZ at birth						- (0.54)
WAZ at wk18		+ (1.00)				
HAZ at wk18	+ (0.98)	+ (1.00)	+ (1.00)	+ (1.00)	+ (1.00)	+ (1.00)
WHZ at wk18	+ (0.70)		+ (1.00)	+ (1.00)	+ (1.00)	+ (0.74)
Exclusive breast feeding until wk18		- (0.80)	- (0.73)	- (0.67)	- (0.77)	
RBP at wk18		+ (0.72)	+ (0.72)	+ (0.59)	+ (0.69)	
Vitamin D at wk18		- (0.32)				
Mannitol at wk12		- (0.73)	- (0.72)	- (0.60)	- (0.71)	- (0.96)
Mannitol at wk24						- (0.85)
ALA at wk12						+ (0.62)
MPO wk12	- (0.59)	- (0.87)	- (0.87)	- (0.80)	- (0.87)	
Expenditure		+ (0.87)	+ (0.87)	+ (0.60)	+ (0.75)	
Mother weight	+ (0.89)	+ (1.00)	+ (1.00)	+ (0.97)	+ (0.99)	+ (0.90)
Mother height		+ (0.52)	+ (0.52)			
Reg1B at wk12		- (0.58)	- (0.53)			
Soluble CD14 at wk18		- (0.66)	- (0.67)		- (0.62)	

Here, “+” and “-” means positive and negative sign of coefficient estimates. Percentage of variables being selected via 100 bootstrapping samples is listed in parenthesis

As shown in Table 3, there were 16 biomarkers chosen at least once, while 17 biomarkers were not selected by any method. Using the BIC selection criterion for tuning parameter, 4 biomarkers were selected by the stepwise regression, 7 by Adaptive LASSO, 8 by MCP, 9 by SCAD, 11 by LASSO, 12 by Elastic Net. Stepwise tended to select too few biomarkers, while LASSO and Elastic Net selected too many biomarkers. The suggested methods, Adaptive LASSO, MCP and SCAD, selected similar sets of biomarkers in the real data analysis. As the validity measure of variable selection methods, the percentage of times for biomarkers being selected were listed in parenthesis, which were obtained through 100 bootstrapping samples. The results showed that all the selected biomarkers, except for Vitamin D at week 18, had over 52% chance of being selected via the bootstrapping, and some biomarkers such as HAZ at week 18 and mother weight had the selection percentages close to 100%.

When comparing the selection results of Adaptive LASSO, MCP and SCAD, four covariates were selected consistently by all the three methods. Among them, HAZ and WHZ at week 18 and mother weight were positively associated with HAZ at one year, while Mannitol at week 12 was negatively associated with the response. The selection results are clinically meaningful, and our findings are similar to that in [8].

Overall, from the variable selection results, EE and systemic inflammation biomarkers, and measures of maternal health were informative of malnutrition. Particularly, nutritional status (HAZ and WHZ) and RBP at week 18, mother weight, and family expenditure were positively associated with HAZ at one year of life, while mannitol and MPO at week 12, and soluble CD14 at week 18 were negatively associated with the outcome. The predictors selected by all methods such as mother weight and HAZ at week 18 indicate the predestination of malnutrition. These results offer a potential explanation for the burden of malnutrition problems in low-income countries, allow early identification of infants at risk, and suggest pathways for intervention.

## Discussion

This study was motivated by the PROVIDE clinical study to evaluate the association between early-stage non-invasive biomarkers and future child growth. The main challenges in practice are 1) the relatively large set of predictors, including both clinical risk factors and biomarkers and 2) some of them are highly correlated. Through simulations of different signal-to-noise sizes and correlation strength, we compared the numeric performance of stepwise regression and several penalized linear regression methods in a simulation setting similar to the clinical data example. For the biomarker data from the PROVIDE study, SCAD, Adaptive LASSO and MCP are recommended due to their performance based on simulations, with relatively large true positive rates, and relatively small false positive rates. To our expectation, the selection result of the real data confirms the observation from simulations. We also explored the selection results by different methods when the SNR and correlation are high or low via simulations. Also, these penalized linear regression methods can be applied to generalized regression models such as Logistic or Poisson regression.

Our study addressed an important question in the field of international health and environmental enteropathy, namely how to analyze large datasets with highly correlated variables or predictors. Identifying the non-invasive biomarkers associated with malnutrition is the first step. The ultimate goal of the EE studies is to have better understanding of underlying pathogenesis and to facilitate the development of treatment strategies for malnutrition. Nevertheless, our findings and suggested methods are not only applicable to the EE studies, but also to the other biomedical studies for biomarker selection.

In this study, we only considered the situation similar to the clinical study where sample size is larger than variable dimension (*n*>*p*). For cases that *p* is at the same scale as *n*, or even *p*≫*n*, Fan and Lv proposed sure independent screening (SIS) to perform variable selection in ultra-high dimension space [26]. The idea is to first perform the correlation learning to reduce dimensionality from high to a moderate scale, and then various variable selection methods can be applied.

Statistical inference is challenging for penalized estimators. Generically, the confidence intervals do not exist for the parameter estimates from penalized methods [27]. Therefore a hypothesis testing cannot be directly established. Tibshirani proposed standard error approximation formula using the bootstrapping method [4]. Fan and Li used sandwich formula to estimate the covariance matrix [5]. However, the approximate covariance matrix by their formula produced an estimated variance 0 for non-selected predictors with $\hat {\beta }_{j}=0$ [28]. The same issue happens when residual bootstrapping is applied. The signs of non-zero components of *β* are estimated correctly with high probability, but the estimators of the zero-components may take both positive and negative values with positive probabilities [28]. Wasserman and Roeder proposed a two-stage procedure for valid inference [29]. In their method, the data is randomly divided into two parts: training and testing datasets. In the training data, penalized linear regression is used to select informative variables as the first stage. In the testing data, ordinary least squares (OLS) is applied to compute standard errors and *p*-values for the variables selected in the first stage. A drawback of the single-split method is that the result may depend on how the data is split. To improve this, Meinshausen et al. suggested multi-split method, which repeats the single-split multiple times, and obtains the empirical distribution of the *p*-values [30]. Recently, Lockhart et al. proposed the covariance test statistic to test the significance of the predictor variable that enters the current LASSO model [31]. Since it is a conditional test, the interpretation of *p*-value is different; given all active variables entering in the LASSO path previously, the *p*-value is for the significance of the next variable entering the model [32]. To our knowledge, significant testing on selected variables methods is still an open problem.

In summary, we assessed the numerical performance of penalized linear regression methods through simulations for correlated covariates or predictors, and further applied the suggested methods to the selection of EE biomarkers in a Bangladesh birth cohort. Our study was motivated by a clinical study, and our findings are readily applicable to other EE studies, or to other biomedical studies with high-dimensional and correlated predictors, for biomarker selection. The strengths of this study are the practicality and applicability of our findings, that is, the plausible application of the penalized regression methods to high-dimensional and correlated data. In the era of big data, it is pivotal to decipher the large and massive data and to retrieve important information from them. We hope that our recommended methods would provide some helpful and practical guide in dealing with such big data. The weakness of the study is the inability to quantify the significance or the relative importance of these biomarkers. As discussed above, significance testing in the penalized regression methods remains challenging. Data mining techniques such as the random forest method would be useful to evaluate the relative importance of the biomarkers.

## Conclusions

Overall, through simulation studies, penalized linear regression methods such as SCAD, Adaptive LASSO and MCP should be considered as plausible alternatives to traditional stepwise regression. In the PROVIDE study, selected predictors such as HAZ at week 18, MPO at week 12, and soluble CD14 at week 18 offer a potential explanation for the burden of malnutrition problems in low-income countries, allow early identification of infants at risk, and suggest pathways for intervention. Our findings and suggested methods are not only applicable to the EE studies, but also to the other biomedical studies for biomarker selection.

## Abbreviations

AIC: Akaike information criterion
ALA: Alpha-1 anti-trypsin
AR: Autoregressive
BIC: Bayesian information criterion
CRP: C reactive protein
CV: Cross validation
EE: Environmental enteropathy
FP: False positive
GCV: Generalized cross validation
HAZ: Height-for-age z-score
ICDDR,B: International Centre for Diarrhoeal Disease Research, Bangladesh
LASSO: Least absolute shrinkage and selection operator
LPS: Lipopolysaccharide
MCP: Minimax concave penalty
MPO: Myeloperoxidase
MRME: Median relative model error
OLS: Ordinary least squares
PROVIDE: Performance of rotavirus and oral polio vaccines in developing countries
RBP: Retinol binding protein
RME: Relative model error
SCAD: Smoothly clipped absolute deviation
SES: Socioeconomic status
SNR: Signal noise ratio
SNPs: Single nucleotide polymorphisms TP: True positive
WHO: World health organization
WHZ: Weight-for-height z-score
